# Mauriac syndrome: a rare complication in patients with type 1 diabetes mellitus

**DOI:** 10.1530/EDM-25-0035

**Published:** 2025-08-04

**Authors:** João Oliveira Torres, Diana Cruz Martins, Alexandra Abegão Matias, Nuno Gião, Eduardo Dutra, Rui Malheiro, Milena Mendes, José Silva-Nunes

**Affiliations:** ^1^ Department of Endocrinology, Diabetes and Metabolism, Hospital de Curry Cabral, Unidade Local de Saúde São José, Lisbon, Portugal; ^2^ Faculdade de Medicina, Universidade de Lisboa, Lisbon, Portugal; ^3^ Nova Medical School/Faculdade de Ciências Médicas, Universidade Nova de Lisboa, Lisbon, Portugal; ^4^ Centro Clínico Académico de Lisboa, Lisbon, Portugal; ^5^ Faculdade de Medicina, Universidade de Coimbra, Coimbra, Portugal; ^6^ Department of Pathological Anatomy, Hospital de Curry Cabral, Unidade Local de Saúde de São José, Lisbon, Portugal; ^7^ Department of Internal Medicine, Hospital de Santo António dos Capuchos, Unidade Local de Saúde São José, Lisbon, Portugal; ^8^ Department of Gastroenterology, Hospital de Santo António dos Capuchos, Unidade Local de Saúde São José, Lisbon, Portugal

**Keywords:** Mauriac syndrome, hepatomegaly, type 1 diabetes mellitus, glycogenic hepatopathy

## Abstract

**Summary:**

Mauriac syndrome is a rare complication in patients with type 1 diabetes. It presents with poor glycemic control and hepatomegaly due to extensive liver glycogen deposition. Whether behavioral or genetic factors play key roles in its pathophysiology remains a subject of debate. We present the case of a 19-year-old woman with poorly controlled type 1 diabetes mellitus and persistently elevated liver enzymes who arrived at the emergency department with diabetic ketoacidosis and hepatomegaly. Blood tests revealed the absence of an associated viral or autoimmune liver disease. Transient liver elastography showed moderate steatosis. Liver biopsy results were consistent with glycogen hepatopathy. Sequencing of genes associated with glycogen storage diseases revealed no pathogenic variants, supporting a non-genetic mechanism for Mauriac syndrome. Insulin regimen and dietary plan were reviewed. Distinction of glycogenic hepatopathy from metabolic dysfunction-associated fatty liver disease is often difficult and frequently only possible through liver biopsy. An accurate diagnosis of Mauriac syndrome carries important prognostic information, as associated hepatomegaly tends to regress through optimization of glycemic control.

**Learning points:**

- Mauriac syndrome is a rare complication of poorly controlled type 1 diabetes, presenting with elevated liver enzymes and hepatomegaly due to extensive liver glycogen deposition.
- Liver biopsy plays a key role in distinguishing glycogenic hepatopathy from metabolic-associated steatotic liver disease.
- Adequate glycemic control often leads to hepatomegaly regression and normalization of liver enzyme levels in Mauriac syndrome.

## Background

In 1930, Pierre Mauriac first described Mauriac syndrome (MS) in children with poorly controlled type 1 diabetes mellitus (T1DM), hepatomegaly, cushingoid features, delayed puberty, and growth retardation (1). Since then, although several clinical case reports of glycogenic hepatopathy have been published worldwide, MS remains underrecognized by physicians (2, 3, 4, 5). It is also known as ‘diabetic glycogenosis’, ‘hepatic glycogenosis’, or ‘glycogen hepatopathy’.

In 2016, MacDonald *et al.* identified a mutation in the glycogen phosphorylase kinase subunit in a patient with T1DM, leading to massive accumulation of glycogen in hepatocytes (6). Although this discovery represents a significant advance in our understanding of the mechanistic causes of MS, its diagnostic and pathophysiological importance remains uncertain.

We describe the report of a 19-year-old woman with persistently poorly controlled T1DM who showed to the emergency department with diabetic ketoacidosis and hepatomegaly and was further diagnosed with MS. We conducted a narrative review of the current literature on MS, with a focus on its pathophysiology and management.

## Case presentation

A 19-year-old woman went to the emergency room with a 5-month history of progressive postprandial vomiting, fullness, and abdominal pain and distension, with diminished oral intake in the previous 48 hours.

She had a history of poorly controlled T1DM diagnosed at 11 years and 8 months of age. She reported glycated hemoglobin persistently higher than 10% and multiple hospital admissions due to diabetic ketoacidosis during the previous 2 years. After one of the admissions for diabetic ketoacidosis, transaminases were temporarily increased: aspartate aminotransferase (AST) 1,218 U/L (reference range (RR): 13–26 U/L) and alanine aminotransferase (ALT) 650 U/L (RR: 9–24 U/L); gamma-glutamyl transferase (GGT) and alkaline phosphatase (AP) were only mildly elevated. At that time, screening was negative for hepatitis A, B, and C, Epstein-Barr, and cytomegalovirus infections. Antibodies for liver autoimmune diseases were negative, and liver ultrasound showed a round liver with 15.5 cm. There was no record of hepatotoxin ingestion. Subsequent follow-up showed normalization of transaminases and persistence of mild elevation of GGT and AP.

She was treated with domperidone 10 mg once a day, pantoprazole 20 mg once a day, and insulin glargine 28 units once a day, insulin aspart 1.5 units per 12 g carbohydrates before each meal plus 1 unit for every 40 mg/dL glycemia above 100 mg/dL. However, she frequently missed preprandial insulin administration.

She experienced menarche at 13 years of age. Her family medical history was unremarkable.

Physical examination revealed tachycardia, Kussmaul breathing, moon facies, tender hepatomegaly with a smooth hepatic border, breast development in Tanner stage M4, and height between percentiles 3 and 15 (inside the family target).

**Figure 1:**
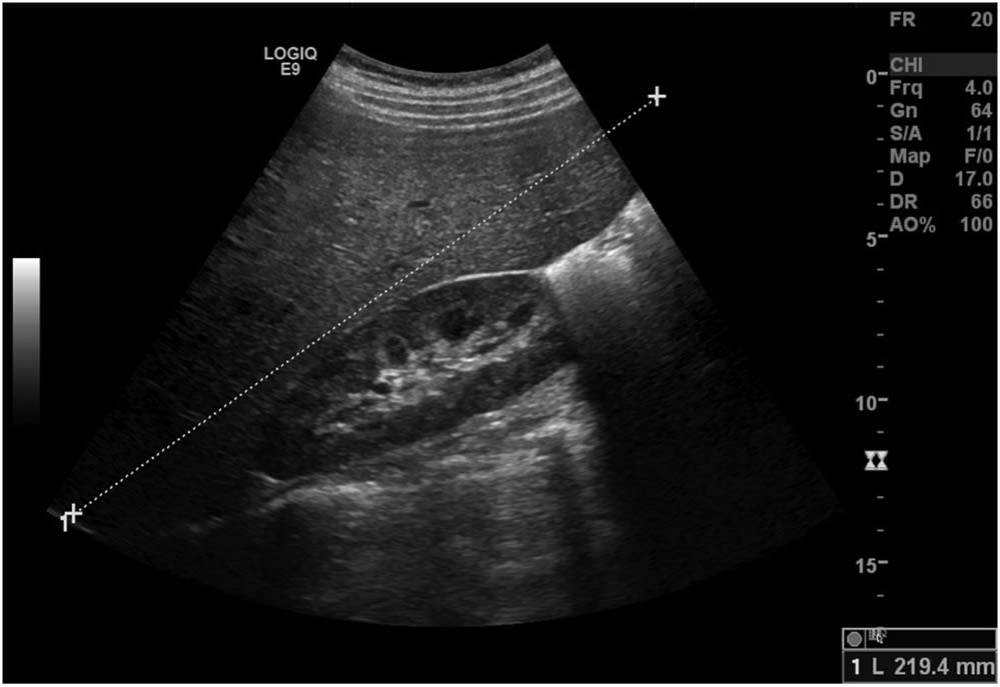
Abdominal ultrasonography revealed a liver without focal lesions, with a round border, homogeneous structure, preserved echogenicity, and increased dimensions (22 cm), causing epigastric swelling.

Laboratory studies unveiled a blood glucose level of 450 mg/dL, metabolic acidosis with a pH of 7.17, HCO3+ of 6.4 mg/dL, serum lactate of 0.6 mmol/L, and point-of-care ketonemia of 7.5 mmol/L. Renal function was preserved, with no significant electrolyte disturbances. The GGT and AP levels were 222 U/L (RR: 9–36 U/L) and 198 U/L (RR: 40–150 U/L), respectively. ALT and AST levels were 66 U/L (RR: 0–55 U/L) and 40 U/L (RR: 5–34 U/L). Prothrombin time and total bilirubin were normal. Abdominal ultrasonography revealed a liver without focal lesions, with a round border, homogeneous structure, preserved echogenicity, and increased dimensions (22 cm), causing epigastric swelling (Fig. 1). There was no evidence of splenomegaly, dilation of the gallbladder, intrahepatic or extrahepatic bile ducts, or ascites in the upper abdominal quadrant.

She was diagnosed with diabetic ketoacidosis and started treatment accordingly. The patient was then admitted for further investigation.

## Investigation

**Figure 2:**
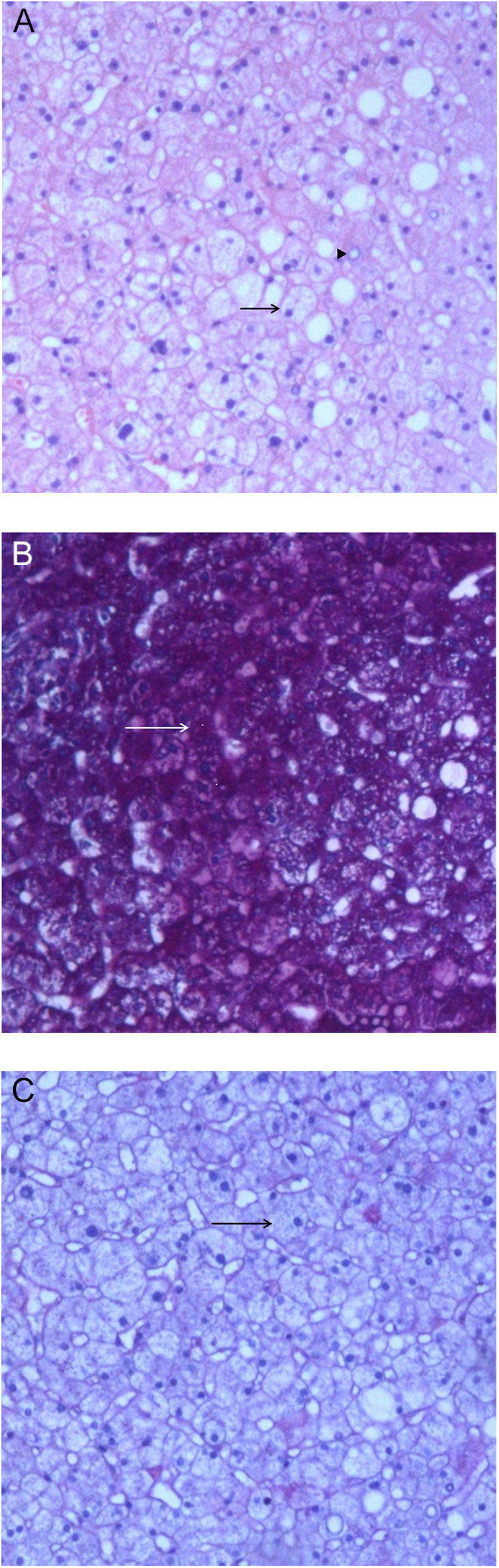
Patient liver biopsy: (A) diffuse hepatocyte enlargement with pale cytoplasm and prominent cytoplasmic membrane (black arrow). Occasional hepatocytes with glycogenated nuclei (triangles) were observed (H&E stain, ×100); (B) Periodic acid–Schiff (PAS) staining showing abundant scattered glycogen in the cytoplasm of hepatocytes (white arrow) (PAS stain, ×100); (C) PAS-D stain digests glycogen (black arrow), supporting the diagnosis of glycogenic hepatopathy (PAS-D stain, ×100).

During hospitalization, screening was negative for hepatitis A, B, C, and E viral infections. The autoimmune panel also tested negative for autoimmune hepatitis, primary biliary cholangitis, and primary sclerosing cholangitis. Alpha-1 antitrypsin and ceruloplasmin levels were normal. Ferritin was 69 ng/mL (RR: 30–349 ng/mL). Magnetic resonance cholangiopancreatography revealed normal liver parenchyma with no bile duct dilation. Transient liver elastography (FibroScanⓇ) revealed moderate steatosis with no liver fibrosis (F0): median liver stiffness 2.8 KPa (interquartile range: 15%), median controlled attenuation parameter 251 dB/m, with 11/11 valid measurements. Two weeks after admission, there was a slight reduction in GGT and AP: GGT 112 U/L (RR: 9–36 U/L) and AP 136 U/L (RR: 40–50 U/L), synchronous with improvement in glycemic control. In order to establish the cause of progressive hepatomegaly and the persistent elevation of GGT and AP, a liver biopsy was conducted. Liver biopsy (Fig. 2) showed hepatocyte enlargement with pale cytoplasm. Occasional hepatocytes with glycogenated nuclei were observed. Periodic acid–Schiff (PAS) staining revealed abundant scattered glycogen in the cytoplasm of hepatocytes. PAS–Diastase (PAS–D) stain digested glycogen, supporting the diagnosis of glycogenic hepatopathy.

Next-generation sequencing of genes associated with glycogen storage diseases was requested (*AGL*, *ADLDOA*, *ALDOB*, *ENO3*, *EMP2A*, *FBP1*, *G9PC*, *GAA*, *GBE1*, *GYG1*, *GYS1*, *LAMP2*, *LDHA*, *NHLRC1*, *PFKM*, *PGAM2*, *PGK1*, *PGM1*, *PHKA2*, *PHKB*, *PHKG2*, *PRKAG2*, *PYGL*, *PYGM*, *SLC2A2*, and *SLC37A4*). No pathogenic variants were detected.

## Treatment

Insulin regimen and dietary plan were reviewed.

## Outcome and follow-up

Outpatient follow-up showed normalization of liver enzymes 1 month after discharge. However, subsequent appointments revealed poor compliance with the medical advice and relapse of GGT and AP elevation.

## Discussion

We describe the clinical case report of a 19-year-old woman with persistently poorly controlled T1DM who showed to the emergency department with diabetic ketoacidosis, persistently elevated GGT and AP, and hepatomegaly of unknown cause. The liver biopsy revealed glycogen deposits inside the hepatocytes with minimal steatosis, and a diagnosis of MS was made. Sequencing of genes associated with glycogen storage diseases revealed no pathogenic variants, suggesting a non-genetic mechanism for MS.

Although MS was first described nearly 100 years ago, and several case reports have been published since then, it has been underrecognized by physicians (3, 7). MS has a mean age for incidence of 23 years, appearing mainly in children and young adults with poorly controlled T1DM. Female gender is slightly more affected (7).

This patient was diagnosed at 19 years of age, 8 years after T1DM diagnosis. She presented with clinical features consistent with MS, including poorly controlled T1DM, cushingoid facies, stalled puberty, hepatomegaly, and elevated liver enzymes. In this patient, the onset of T1DM after the beginning of puberty might have stalled puberty progression rather than inducing a delay in its onset. Cushingoid features and stalled puberty might be related to the release of counter-regulating hormones, such as cortisol, during hypoglycemic episodes frequently observed in poorly controlled patients with T1DM (3).

The hepatic uptake of glucose occurs through the passive diffusion transporter glucose transporter 2 (GLUT-2). Therefore, hyperglycemia translates to a high glucose flux to hepatocytes in an insulin-independent manner. When liver glucose levels increase above 90 mg/dL, glucose is converted into glucose-6-phosphate by glucokinase and then into glycogen by glycogen synthase. Glucose and insulin promote glycogen synthase activity. On the other hand, glycogenolysis occurs through glycogen phosphorylase, an enzyme activated by glycogen phosphorylase kinase in the presence of epinephrine and glucagon; this process is inhibited by high glucose levels. The hepatomegaly observed in MS results from excessive glycogen accumulation within hepatocytes (4, 6).

Recently, MacDonald *et al.* discovered an inactivating mutation in the catalytic subunit of glycogen phosphorylase kinase (PHKG2 G>A) in a patient with MS. The authors proposed a new mechanistic cause for MS, where the referred heterozygous mutation and hyperglycemia-induced inhibition of glycogen phosphorylase block glycogenolysis and lead to a massive hepatomegaly (6).

However, our patient did not harbor any pathogenic variants of the 26 genes associated with glycogen storage diseases, including the *PHKG2* gene. Therefore, we conclude that this mutation is not a sine qua non condition for MS development. In fact, this clinical case report rather supports a behavioral hypothesis, where the concurrent hyperglycemia and erratic insulin administration led to excessive glycogenesis and hepatomegaly. The reason why only a few patients with poorly controlled T1DM develop MS remains unknown. There are also some reports of glycogenic hepatopathy occurring in patients with post-gastric bypass dumping syndrome, short-term high-dose corticosteroid therapy, and insulin overdose, whose mechanism of disease also involves hyperglycemia and hyperinsulinemia. In addition, glycogenic hepatopathy was also reported in patients with anorexia nervosa, possibly as an adaptive response to protect from fatal hypoglycemia. It has also been reported in patients with type 2 diabetes, the mechanism of which is still unknown (7).

Patients usually present with abdominal pain, nausea, vomiting, and elevated liver enzymes. Recently, Khoury *et al.* proposed a diagnostic algorithm for MS (4). In the presence of elevated liver enzyme levels, the first step should be to exclude any viral, autoimmune, or metabolic liver disease. Blood tests frequently show hepatocellular rather than cholestatic injury, although the latter may be the predominant feature, as in our patient (4, 5, 8). Elevated liver enzyme levels in patients with diabetes are most frequently caused by metabolic dysfunction-associated steatotic liver disease (MASLD).

The distinction between MASLD and glycogen hepatopathy is often difficult by imaging studies and may require liver biopsy. A dual-echo magnetic resonance imaging (MRI)/computed tomography (CT) may be able to distinguish these two clinical entities (4, 7). CT scan may show increased liver density due to glycogen deposition in MS, whereas MASLD is associated with reduced liver density (7). T2-weighted MRI shows a high-intensity liver in MS and a low-intensity liver in MASLD. On gradient dual-echo MRI, the MS liver appears isointense between in-phase and out-of-phase images. The MASLD liver, however, has a low intensity on the in-phase, and a high intensity on the out-of-phase image (7). If imaging studies support the diagnosis of MS, optimal glucose control should be achieved. If there is no improvement after 1–3 months, liver biopsy should be considered (4, 7). There is no role for transient liver elastography in the distinction of glycogen hepatopathy from MASLD (3, 4).

In MS, liver biopsy shows marked glycogen accumulation leading to pale, swollen hepatocytes, absence or minimal fatty change and inflammation, and preserved architecture, with no significant fibrosis. PAS staining is positive for glycogen deposits that are digested with PAS–diastase (4, 5, 7). Our patient’s liver biopsy showed all those features.

In MS, good glycemic control often leads to hepatomegaly regression and normalization of liver enzyme levels within weeks to months. Therefore, accurate differentiation between MS and MASLD carries significant management and prognostic information, as the latter may progress to hepatic fibrosis, irrespective of glycemic control (3, 4, 7). Patient compliance with medical advice and adherence to the therapeutic measures are, therefore, key elements in the prognosis of MS.

To conclude, MS is a frequently underrecognized complication in patients with T1DM. Although a mutation in PHKG2 has been described as a mechanistic cause for this syndrome, poor glycemic control with erratic high-dose insulin administration remains the most widely accepted mechanism for its development. An adequate diagnosis of the syndrome and an effective therapeutic education provide greater therapeutic and prognostic value for these patients.

## Declaration of interest

The authors declare that there are no conflicts of interest that could be perceived as prejudicing the impartiality of the research reported.

## Funding

This research did not receive any specific grant from any funding agency in the public, commercial, or not-for-profit sector.

## Patient consent

Written informed consent for publication of their clinical details and clinical images was obtained from the patient.

## Author contribution statement

JOT, DCM, AAM, NG, ED, RM, and MM were responsible for direct clinical work. JOT and AAM conducted the investigation. JOT wrote the original draft. DCM and MM contributed to conceptualization. DCM and RM provided supervision. DCM, AAM, NG, ED, RM, MM, and JSN participated in writing, reviewing, and editing. NG and ED contributed to visualization. JSN managed the project.
